# Supplementary material for: Benzalkonium Chloride Induces a VBNC State in Listeria monocytogenes
Source: Microorganisms. 2020 Jan 28;8(2):184. doi: 10.3390/microorganisms8020184 (PMC7074807; doi:10.3390/microorganisms8020184)
Supplement: Supplementary file 1 [file microorganisms-08-00184-s001.pdf]

Supplementary Figure to

Benzalkonium chloride induces a VBNC state in *Listeria monocytogenes*

Running title: **BC induces VBNC in *L. monocytogenes***

Matthias Noll[1]#, Katharina Trunzer[1], Antje Vondran[1], Szilvia Vincze[2], Ralf Dieckmann[2], Sascha Al Dahouk[2] & Carolin Gold[1]

[1]Institute for Bioanalysis, Department of Applied Sciences, Coburg University of Applied Sciences and Arts, Friedrich-Streib-Straße 2, D-96450 Coburg, Germany.

[2]German Federal Institute for Risk Assessment, Max-Dohrn-Str. 8-10, 10589 Berlin, Germany

#Corresponding author: Mailing address: Institute for Bioanalysis, Department of Applied Sciences, Coburg University of Applied Sciences and Arts, Friedrich-Streib-Straße 2, D-96450 Coburg, Germany

Phone: +49-9561-317645, Fax: +49-9561-317346; E-mail: matthias.noll@hs-coburg.de

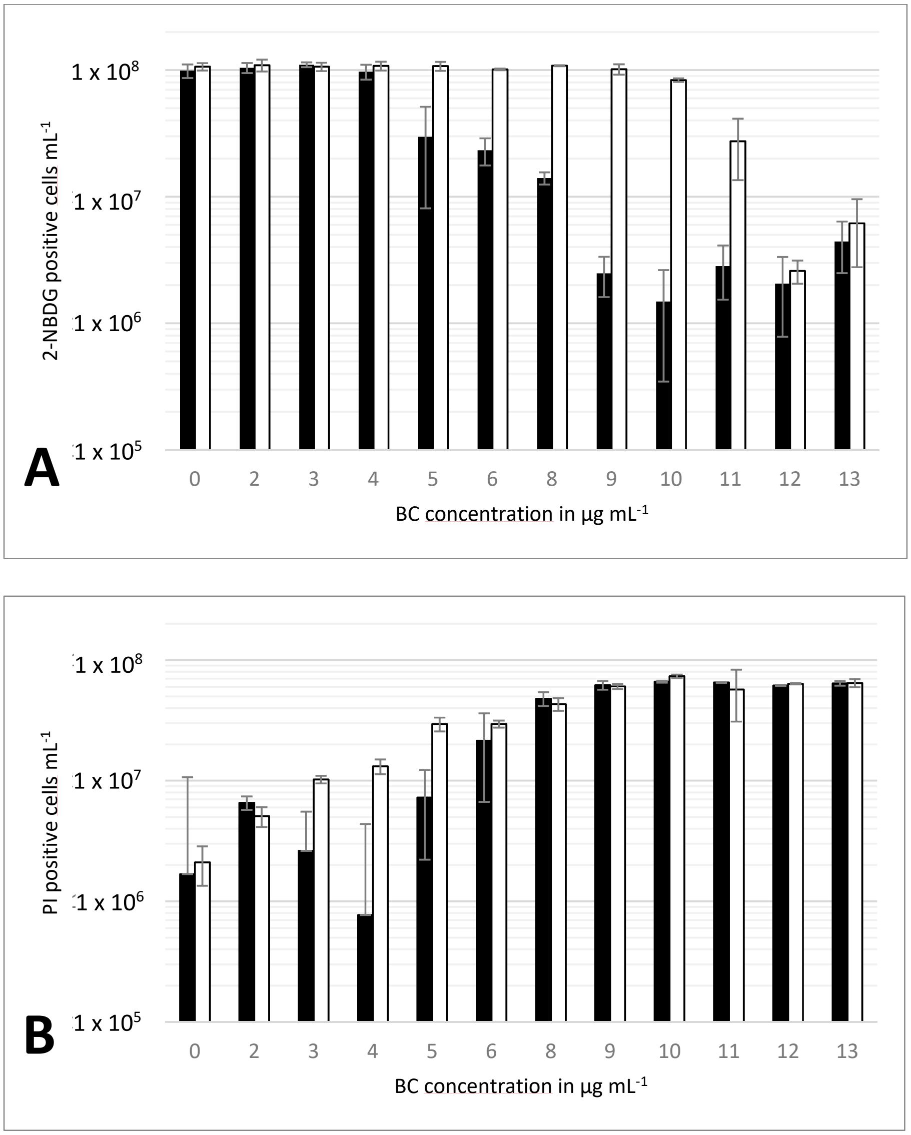

Supplementary Figure. 1 Mean cell counts of PI positive (A) and 2-NBDG positive (B) cells for parent (black bars) and adapted (white bars) *L. monocytogenes* SLCC2540 following cultivation in BHI containing benzalkonium chloride (BC) in various concentrations. Error bars indicate standard deviation of three replicates.
